# Fluid shear stress impacts ovarian cancer cell viability, subcellular organization, and promotes genomic instability

**DOI:** 10.1371/journal.pone.0194170

**Published:** 2018-03-22

**Authors:** Alexandra R. Hyler, Nicolaas C. Baudoin, Megan S. Brown, Mark A. Stremler, Daniela Cimini, Rafael V. Davalos, Eva M. Schmelz

**Affiliations:** 1 School of Biomedical Engineering and Sciences, Virginia Tech - Wake Forest University, Blacksburg, VA, United States of America; 2 Department of Biological Sciences and Biocomplexity Institute, Virginia Tech, Blacksburg, VA, United States of America; 3 Department of Human Nutrition, Foods and Exercise, Virginia Tech, Blacksburg, VA, United States of America; 4 Department of Biomedical Engineering and Mechanics, Virginia Tech, Blacksburg, VA, United States of America; The University of Hong Kong, HONG KONG

## Abstract

Ovarian cancer cells are exposed to physical stress in the peritoneal cavity during both tumor growth and dissemination. Ascites build-up in metastatic ovarian cancer further increases the exposure to fluid shear stress. Here, we used a murine, *in vitro* ovarian cancer progression model in parallel with immortalized human cells to investigate how ovarian cancer cells of increasing aggressiveness respond to $<1\frac{dyne}{cm^{2}}$ of fluid-induced shear stress. This biophysical stimulus significantly reduced cell viability in all cells exposed, independent of disease stage. Fluid shear stress induced spheroid formation and altered cytoskeleton organization in more tumorigenic cell lines. While benign ovarian cells appeared to survive in higher numbers under the influence of fluid shear stress, they exhibited severe morphological changes and chromosomal instability. These results suggest that exposure of benign cells to low magnitude fluid shear stress can induce phenotypic changes that are associated with transformation and ovarian cancer progression. Moreover, exposure of tumorigenic cells to fluid shear stress enhanced anchorage-independent survival, suggesting a role in promoting invasion and metastasis.

## 1 Introduction

All cells exist in a physiologic environment that is determined by chemical and physical factors; in concert, these factors direct tissue growth, organization and function but also can cause or contribute to diseases such as cancer. Indeed, it has been suggested that different stresses arises in the cellular microenvironment can, in concert with changes arising within a cell’s genome, contribute to chromosomal instability-mediated cancer evolution [1] However, while there have been tremendous efforts to characterize the cellular and molecular compositions of the tumor microenvironment and their contributions to cancer development and progression, the full impact of physical stimuli remain incompletely characterized.

Epithelial ovarian cancer (EOC) is the fourth most deadly cancer, with a 5-year survival rate below 30% when diagnosed after the cancer has spread beyond its boundaries [2, 3]. During metastasis, ovarian cancer cells exfoliate from the primary tumor and disseminate throughout the peritoneal cavity, a distribution process supported by fluid motion [4, 5]. These disseminating single tumor cells or cell clusters [6] can adhere to the organs in the peritoneal cavity and initiate secondary tumor outgrowth [7]. Ovarian cancer cells exfoliated into the peritoneal cavity are exposed to shear and tensile stresses and pressure from solid tumor formation and ascites build-up. Specifically, continual fluid shear stress (FSS) is imposed onto the cells due to gastrointestinal and diaphragm movements, abdominal pressure changes, gravity, and, importantly, ascites build-up in advanced stages of ovarian cancer [5, 8]. Thus, the magnitude of exposure to FSS is dependent on the individual increase of ascites volume in the peritoneal cavity of women with ovarian cancer. These biomechanical forces induce rapid signaling events from the extracellular environment through the membrane into the cytosol and the nucleus. This process, termed mechanotransduction, elicits cellular responses that impact cell proliferation, cytoskeleton remodeling, adhesion, migration and other cancer cell characteristics [4, 9, 10]. Furthermore, the biomechanical properties of the cancer cells themselves change during progression [11–14], enabling the cells to adapt to their changing microenvironment, and migrate, adhere and invade at distant sites.

While the exact patterns of fluid motion within the peritoneal cavity remain unknown, the diaphragm and organ movements are expected to generate flows that remain in the laminar regime. No measurements of FSS magnitude in the peritoneal cavity are available. However, the magnitude of force on cells in the human peritoneal cavity is estimated to be on the order of $0-10\frac{dyne}{cm^{2}}$ for physiological shear stress based upon measurements in pig ileum, the only *in vivo* measurements taken related to FSS and peritoneal organs [4, 5, 15, 16]. Since peritoneal flow is not driven by high-pressure contractions and is in a large volume space, it is reasonable to estimate that the maximum FSS values would be comparable to the slower velocity flows in venous arteries, which have been measured in humans to remain below $5\frac{dyne}{cm^{2}}$ [17]. The fluid motions in the peritoneal cavity are highly variable from woman to woman due to differences in body size, peritoneal fluid volume, adipose tissue volume and diaphragm movement making direct measurements difficult. Short-term exposure to low magnitudes of FSS has been shown to impact neoplastic progression of established cell lines [4, 18]. The effect of long-term exposure to FSS (more than two passages) as well as the differential response of benign cells, early and late stages of the disease and the impact of biophysical stimuli on disease progression are unknown.

The evaluation of the impact of FSS on EOC and the determination of the molecular events triggered by FSS-activated mechanotransduction require cancer models and dynamic testing platforms with increased efficiency in order to translate findings related to cancer diagnosis and progression to clinical outcomes. Specifically, long-term evaluations of mechanical stimuli on EOC have been hindered by a lack of cell models that allow for studying the dynamic progression of cancer. Here we use our spontaneously transformed mouse ovarian cancer epithelial cell (MOSE) model that dynamically progresses from a premalignant, non-tumorigenic state to a transitional and highly aggressive malignant phenotype in *in vitro* culture [19–21]. In addition, we use immortalized, benign (OCE1) and immortalized, carcinogenic (SKOV-3) human cell lines. As such, the MOSE model enables long-term studies of cellular and molecular changes in defined cancer stages, allowing for identification of triggering events and for a more comprehensive understanding of ovarian cancer progression. In parallel, the two stages of human disease allow confirmation that our mouse model accurately captures human cancer progression.

We hypothesized that even relatively small values of fluid-flow induced shear stress will differentially affect the various stages of disease. To test this hypothesis, we imposed a slow, swirling motion onto MOSE, OCE1, and SKOV-3 cells to mimic the fluid movement in the peritoneal cavity. We found that the resulting FSS of magnitudes $<1\frac{dyne}{cm^{2}}$ affected cell viability, spheroid-forming capacity, and cytoskeleton organization. Additionally, the imposed FSS induced chromosome numerical changes in benign cells, suggesting a potential impact of FSS on tumor initiation and progression.

## 2 Materials and methods

### 2.1 Cell lines and culture methods

MOSE cell lines, representing progressive stages of ovarian cancer, were developed from C57BL/6 mice as previously described [19, 22]. The genotype and phenotype of these cells has since been extensively characterized to verify parallels to human disease progression [11, 23–27]. For the present studies, we employed the non-tumorigenic MOSE-E and the tumorigenic (slow-developing disease) MOSE-L. To generate the highly aggressive MOSE-L_TIC*v*_ (fast-developing disease), syngeneic MOSE-L cells were injected intraperitoneally into C57BL/6 mice and harvested via peritoneal lavage after 4 to 6 weeks to select for a more aggressive phenotype [7]. These MOSE tumor-initiating cell variants (TIC*v*) were further transduced with firefly luciferase lentiviral particles (GeneCopoeia, Rockville, MD) to facilitate *in vivo* imaging of cancer cell outgrowth. MOSE cells were routinely cultured in high glucose Dulbecco’s Modified Eagle’s Medium (DMEM, Sigma-Aldrich, St. Louis, MO), supplemented with 4% fetal bovine serum (Atlanta Biological, Norcross, GA) and 100*μg*/mL of each penicillin and streptomycin.

SKOV-3 cells (ATCC^®^, HTB77^™^), human ovarian epithelial carcinogenic cells, were purchased from ATCC (American Type Culture Collection, Manassas, VA). SKOV-3 cells were cultured in high glucose DMEM (Sigma-Aldrich, St. Louis, MO) supplemented with 10% fetal bovine serum (Atlanta Biological, Norcross, GA) and 100*ng*/*mL* each of L-glutamine (Gibco, Thermo Fisher Scientific, Waltham, MA), sodium pyruvate (Gibco, Thermo Fisher Scientific, Waltham, MA), and MEM Non-Essential Amino Acids (Gibco, Thermo Fisher Scientific, Waltham, MA).

OCE1 cells, benign ovarian surface epithelial cells immortalized with human telomerase reverse transcriptase (hTERT), and FOMI medium were purchased from the Live Tumor Culture Core (LTCC) at the University of Miami, Sylvester Comprehensive Cancer Center (http://sylvester.org/shared-resources/live-tumor-culture-core) [28]. OCE1 cells were cultured in FOMI culture medium supplemented with 25 ng/mL of cholera toxin in BD Primaria culture flasks (BD Biosciences, Franklin Lakes, NJ) [28]. All cells were cultured at 37°C in a humidified atmosphere with 5% CO_2_.

### 2.2 Experimental setup

**Fig 1:**
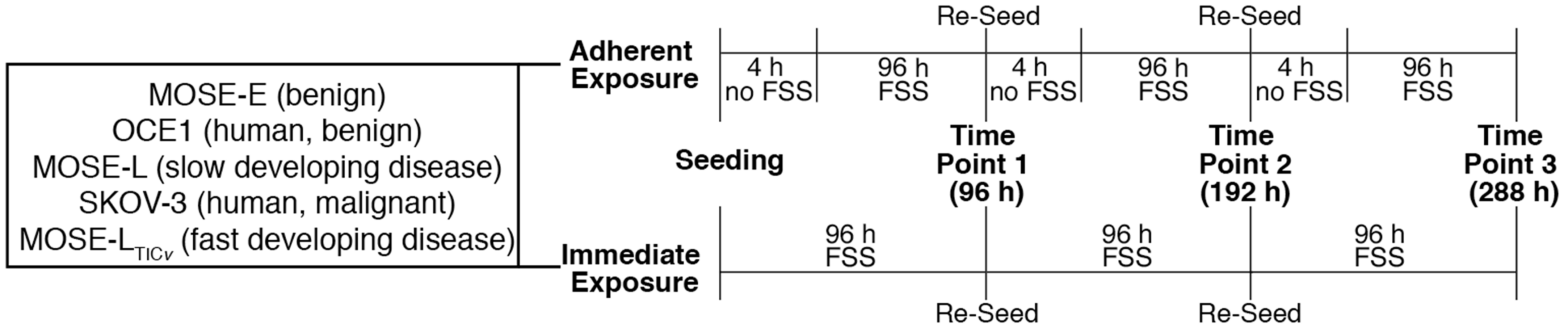
Experimental design. Overview of the experimental design used in this study. Cells were seeded and either immediately placed on the rotator **(A)** or allowed to adhere for 4 h before being placed under FSS conditions **(B)**. Cells were re-seeded at their original density after each 96 hour period of exposure to FSS. Analysis was performed at three different time points corresponding to a total of 96 h, 192 h, or 288h FSS exposure, respectively.

Cells were seeded onto 100mm tissue culture dishes at a density of 2*x*10^5^ cells per dish for all experiments. Cells were either immediately placed (named -imm- or -immediate- throughout the manuscript) onto a Lab Line Maxi Rotator 4631 (Lab Line, Waltham, MA) or allowed to adhere for 4 hours under static conditions (named—adh- or -adherent-) before placement onto the rotator under regular tissue culture conditions as stated above. The Lab Line rotator moved in both horizontal and vertical directions through a 4.5° angle at a frequency of 10 rpm to generate continual motion of the culture medium and hence induce shear stress on the cells during incubation. We selected this system over parallel flow systems since the current understanding of peritoneal fluid motion predicts swirling and rotating fluid motion and not steady, uni-directional flow fields [5]. Control cells were incubated under static conditions. A summary of the experimental plan is shown in Fig 1.

### 2.3 Estimation of experimental shear stress

The motion of our lab rotator was similar to, but less vigorous than, the “see-saw” motion modeled by Zhou et al. [29]. That model is expected to over-estimate the shear stress produced in our system [30], so we applied the model in [29] to ensure that the shear stress produced in our experiments remained within a low regime and physiological range.

After verifying the FSS values obtained by Zhou et al. by replicating the equation results for their system, we then approximated the shear stress on cells at the center of the culture dish to have a maximum value

$$\tau =\frac{3\pi \mu \theta_{max}}{4T{\left(\frac{h_{0}}{D}\right)}^{2}},$$ (1)

where *μ* is the medium viscosity, *θ*_*max*_ is the maximum deflection angle (4.5° = 0.0785 radians), *T* = 6 s is the rocking time period, *h*_0_ is the initial fluid height and *D* is the dish diameter. *θ*_*max*_ and *T* are based upon the manufacturer specifications of the lab rotator and are held constant throughout this work. We measured the viscosity of DMEM to be $\mu =7.8\times 10^{-3}\frac{dyne*s}{cm^{2}}$ at 37°C. The inner diameter of the culture dishes were 85mm (as measured) and each dish contained 20*mL*(±0.5*mL*) of medium, giving *h*_0_ = 3.5 ± .06mm. Therefore, the characteristic shear stress on cells at the center of the dish was estimated using Eq (1) to be $0.14\frac{dyne}{cm^{2}}$. Given the fluid medium variations, the characteristic shear stress was estimated to be within the defined range of $0.13-0.32\frac{dyne}{cm^{2}}$ for a majority (84–94%) of the dish [29]. Thus, the results reported here were obtained under a relatively low but still physiologically relevant range of FSS since current estimates for the peritoneal cavity are below $5\frac{dyne}{cm^{2}}$ [4, 5, 15–17].

### 2.4 Cell viability and spheroid size tracking

After 96h, representative images at both the center and edges of each 100mm culture dish were taken to monitor and measure spheroid growth using a 2M series Nikon digital camera attached to an inverted Eclipse TS 100 Nikon microscope with a 10x objective (Nikon Instruments, Inc., Melville, NY). Next, cells suspended in the supernatant were collected and all cells -both adherent and spheroids from the supernatant- were trypsinized. Cells were then stained with Trypan Blue (Sigma Aldrich, St. Louis, MO) and counted with a hemocytometer to determine the number of viable cells before re-seeding at the initial seeding density for successive 96-hour periods (3 in total). This experimental setup allows for the long-term exposure of growing and dividing cells to FSS while also allowing the enrichment of cells with FSS-induced altered phenotypes. Spheroid diameters were measured using NIS Elements AR software (Nikon Instruments, Inc., Melville, NY). All cell count data and spheroid diameter measurement data presented are means ± SEM of at least two biological replicates each performed in triplicate.

### 2.5 Fluorescence staining and focal adhesion quantitation

After being passaged twice under FSS conditions, each cell line was seeded at a density of 2 × 10^5^ cells per dish onto 100mm dishes containing several sterile glass coverslips and incubated at either static control or adherent FSS conditions for 96 h. The cells were fixed with 3% paraformaldehyde in 250*mM* HEPES for 10 min, then permeabilized with 0.25% Triton X-100 in 6% paraformaldehyde and quenched with 50*mM* glycine for 10 min.

For actin staining, cells were blocked in 2% chicken serum in PBS for 30 min and then incubated with Alexa Fluor488 conjugated phalloidin (MolecularProbes, Eugene, OR). For CREST and vinculin staining, cells were blocked in 2% chicken serum in PBS for 30 min, incubated with anti-centromere/CREST antibody (Antibodies Inc., Davis, CA) or vinculin primary antibody (Sigma Aldrich, St. Louis, MO), overnight at 4°C, and incubated at room temperature with an Alexa Fluor488 or Alexa Flour598 conjugated secondary antibody (Molecular Probes, Eugene, OR). All coverslips were mounted with Prolong Gold anti-fade mounting medium containing DAPI (Invitrogen, Carlsbad, CA) to visualize nuclei. A Nikon 80i epifluorescence microscope equipped with UV, FITC, and TRITC filters and a DS-U2 monochromatic camera utilizing NIS Elements BR 3.0 software (Nikon Instruments, Inc., Melville, NY) was used for image capture of actin staining. A swept field confocal system (Prairie Technologies, WI, USA) on an inverted epifluorescence microscope (Eclipse Ti, Nikon Instruments, Inc., Melville, NY) equipped with a Lumen 200PRO fluoresence illumination system, a 60x/1.4 NA Plan-Apochromatic contrast objective lens, automated ProScan stage (Prior Scientific, Cambridge, UK), HQ2 CCD camera (Photometrics, Tucson, AZ), and NIS Elements AR software (Nikon Instruments, Inc., Melville, NY) was used for image capture of DAPI-stained nuclei and CREST immunostaining. Adobe Photoshop^®^ was used for all image processing. All CREST and multi-lobed nuclei data were collected by counting at least 1000 cells per coverslip, and these data are presented as the mean ± SEM of at least two, independent replicates. All vinculin data were collected by counting at least 30 cells per coverslip, and these data are presented as the mean ± SEM of at least two, independent replicates.

### 2.6 Metaphase spreads

After the third round of FSS exposure, MOSE-E control and MOSE-E_adh_ cell cultures were incubated in 0.1*μg*/mL colcemid (Karyomax, Invirtrogen) at 37°C for 4-5 h to enrich the population of mitotically arrested cells. The cells were trypsinized and centrifuged at 1,000 rpm for 5 min. Hypotonic solution (0.075 M KCl) was added drop-wise to the cell pellet and incubated for 23 min at 37°C. Then, cells were fixed in 3:1 methyl alcohol:glacial acetic acid and centrifuged 5 min at 1,200 rpm. Fixing was repeated two more times, and then cells were dropped onto slides and air dried. Slides were then stained with DAPI (Molecular Probes, Eugene, OR) and mounted using glass coverslips and anti-fade mounting medium containing 20 mM Tris, 90 % glycerol, and 0.5% N-propyl gallate. Stained slides were imaged with the same swept field confocoal system described in the previous section. Chromosome counts were performed using NIS Elements AR software (Nikon Instruments, Inc., Melville, NY). All metaphase count data were obtained from at least two independent replicates (where at least 35 cells were counted per replicate) and are reported as chromosome number per cell (mean ± SEM).

### 2.7 Statistical analysis

When two groups were compared, an unpaired t-test, a chi-square (*χ*^2^), or a Fisher’s exact test was performed, depending on the type of data analyzed. For multiple group comparisons, one-way analyses of variance (ANOVA) were conducted. In general, a p-value < 0.05 was considered significant. Where significance existed for one-way ANOVAs, post-hoc student’s t-test and Tukey’s analyses were conducted to determine differences between groups. All statistical analyses were conducted using JMP^®^ Pro software (v 11.0.0, SAS Institute Inc., Cary, NC) or Prism software (v 6.0e, Graphpad Software, Inc., La Jolla, CA).

## 3 Results

### 3.1 Shear stress impacts cell viability

In order to begin investigating the impact of FSS on cell viability as a function of disease stage, cells representing benign (OCE1, MOSE-E), slow (MOSE-L) and fast-developing (MOSE-L_TIC*v*_) and human (SKOV-3) disease states were subjected to FSS either immediately upon seeding or after adherence to the plates. Immediate exposure without adherence to the dish aimed to mimic cells already disseminated into the peritoneal cavity where they are exposed to FSS as they exfoliate and metastasize. In contrast, adherence to the culture dish before exposure aimed to mimic cells adhered to the original tumor location or the secondary sites that are exposed to FSS across attached surfaces. There were no significant differences in cell numbers for each of the control groups, grown under static conditions, throughout the duration of the study. It is important to note that the doubling time for each cell type is different (from > 35 h for benign and human cells to 12 h for more malignant murine cells) [19, 28] and, therefore, the cell numbers are higher in the MOSE-L and MOSE-L_TIC*v*_ control groups.

**Fig 2:**
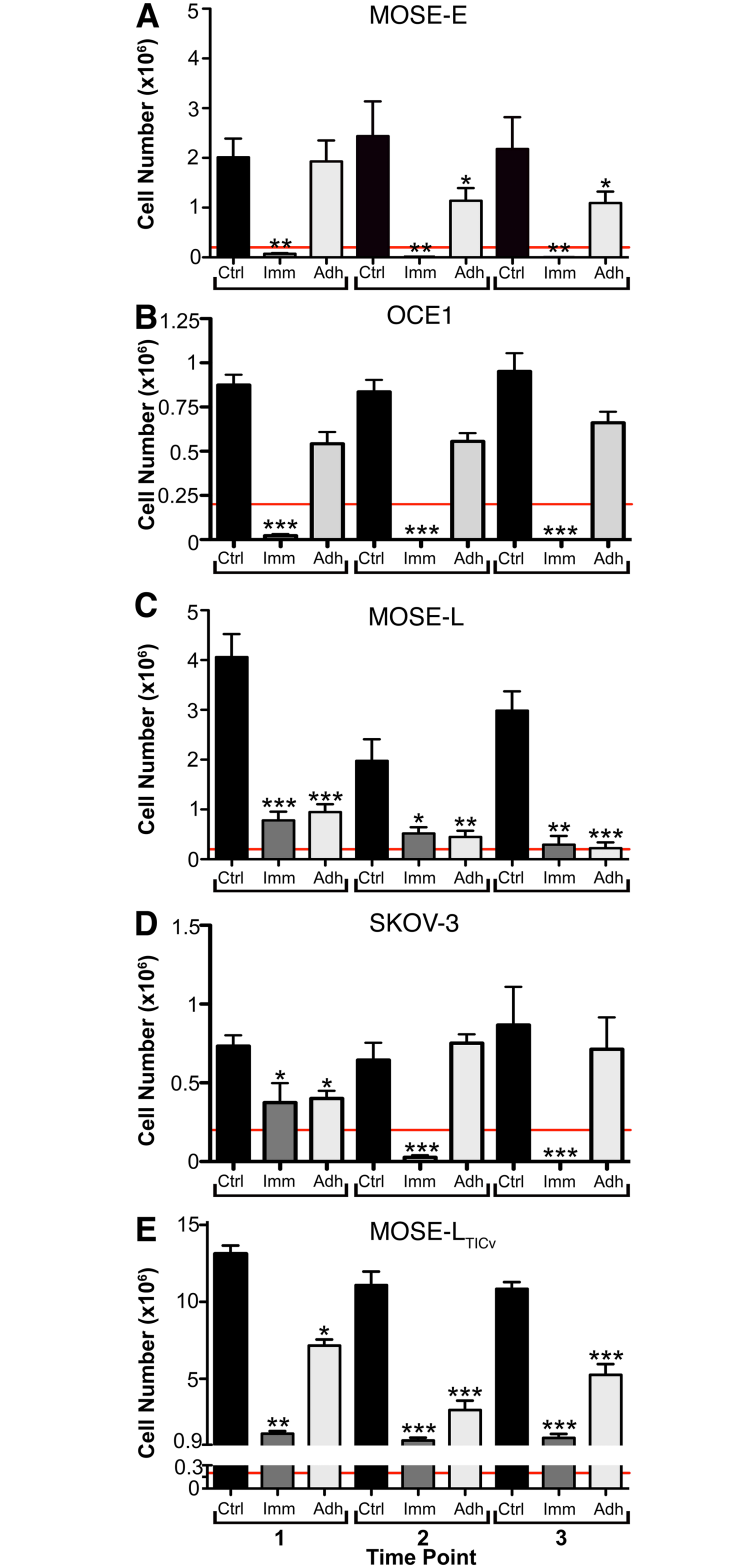
Cell survival. FSS differentially affects cell viability in ovarian cancer cells of different disease stage. Time-dependent changes in average cell number (x10^6^) ± SEM of MOSE-E (**A**), OCE1 (**B**), MOSE-L (**C**), SKOV-3 (**D**), and MOSE-L_TIC*v*_ (**E**) cells subjected to FSS of $\approx 0.14\frac{dyne}{cm^{2}}$ for 96 h periods (time points 1-3) are shown. In each graph, the red line indicates the initial seeding number (2x10^5^). Asterisks denote statistical significance (ANOVA, Tukey’s * p< 0.05, ** p< 0.005, and *** p< 0.001) as compared to the corresponding control group at the same time point.

Immediate exposure to FSS resulted in a significant reduction in cell number in all cell lines compared to the control group at all three time points (each 96 h exposure to FSS), indicating a sensitivity to continual shear stress conditions. The benign MOSE-E_imm_ and OCE1_imm_ cells were the most sensitive, with a reduction in cell number below initial seeding density (Fig 2A and 2B) indicating a net loss of cells. Similarly, MOSE-L_imm_ and SKOV-3_imm_ cells were highly sensitive to FSS, and after exposure to FSS for three passages (96 h each), their number was at or lower than their initial seeding density (Fig 2C and 2D). In contrast, the MOSE-L_TIC*v*-imm_ initially showed a significantly reduced number of viable cells at the first point, but this lower cell number was maintained at subsequent time points (Fig 2E), suggesting a lack of immediate adaptation to the effects of FSS (i.e., expansion of resistant sub-populations) but also a lack of cumulative FSS effects. When the cells were allowed to adhere before exposure to FSS, the reduction of cell number was less severe than the immediate groups; only the MOSE-L_adh_ showed a similar reduction in the number of viable cells as in the group immediately exposed to FSS (Fig 2C). This less severe reduction in cell number suggests that either the cells can adapt to the FSS, or this experimental design with successive re-seeding selects for more resistant sub-populations.

### 3.2 Shear stress induces growing spheroid formation

In the peritoneal cavity, exfoliated tumor cells disseminate as single cells or as aggregates (spheroids). The aggregation of tumor cells enhances their survival and invasive capacity [31–33]; thus, spheroid formation may reflect a more metastatic phenotype. Here, we monitored spheroid formation at all three time points (each 96 h period of exposure to FSS, see Fig 1) in response to physiological magnitudes of FSS.

**Fig 3:**
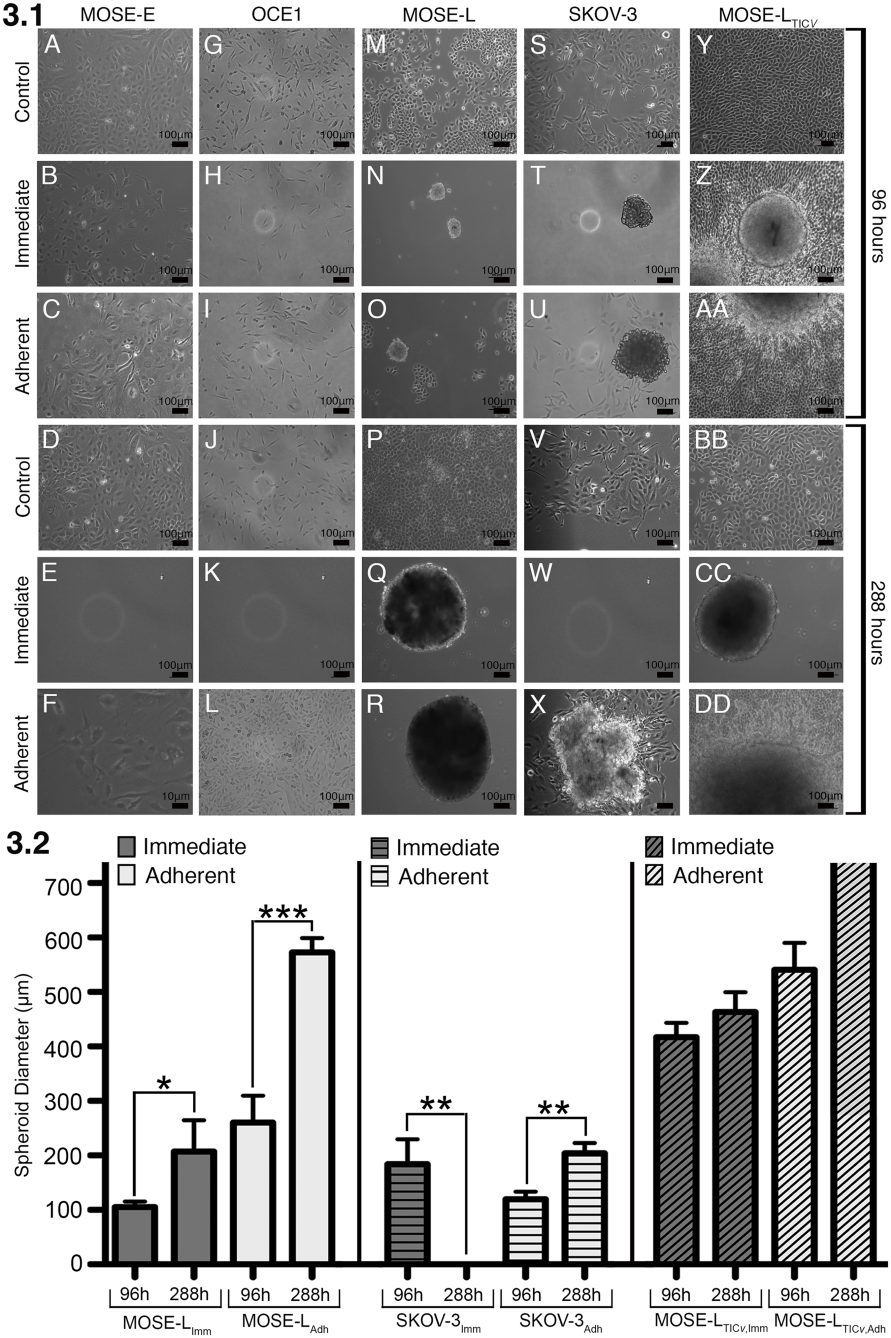
Spheroid formation images and size quantification. FSS induces spheroid formation in tumorigenic ovarian cancer cells. (**3.1**) Images of differential spheroid formation, adherence and outgrowth of benign (MOSE-E, OCE1), tumorigenic (MOSE-L, SKOV-3), and highly aggressive (MOSE-L_TIC*v*_) ovarian cancer cells at 96 h (A-I) and 288 h (time point 3, K-S) in response to FSS. All representative images were taken at the center of the plates. (**3.2**) the diameter of the formed spheroids were measured and averaged at each time point to monitor growth over time. Significant growth in spheroid diameter was measured in both FSS-exposed MOSE-L cells. In addition, MOSE-L_TIC*v*-imm_ cells formed large spheroids that grew over time. Note: the MOSE-L_TIC*v*-adh_ cells re-attached to the culture dishes with an adherent monolayer outgrowth too large to be measured, but the diameters after 288 h are at least 700μm. Asterisks denote statistical significance (t-test, * p< 0.05, ** p< 0.005, and *** p< 0.001).

No spheroid formation was observed in any of the control groups throughout the duration of the study (Fig 3 Rows 1, 4). The benign MOSE-E and OCE1 cells did not form spheroids under either FSS condition, confirming our previous reports that spheroid formation is limited to tumor-forming cells while benign cells do not have this capacity [19]. Very few viable, adherent benign MOSE-E_imm_ (Fig 3B) and OCE1_imm_ (Fig 3H) cells remained after exposure to FSS for 96 h (time point 1) but none were detected after three passages (time point 3) with continuous exposure to FSS (Fig 3E and 3K). However, at the time point 3, viable cells were still apparent in the adherent groups (Fig 3F and 3L).

In contrast, the tumorigenic cells lines responded to FSS by detaching and forming aggregates or spheroids. These spheroids were initially small under both treatment conditions (Fig 3N, 3O, 3T and 3U). However, after repeated passaging that included the trypsinization of these spheroids and re-seeding of single cell solutions at the original cell density, spheroids formed by MOSE-L_imm_ and MOSE-L_adh_ cells grew in diameter from 105 ± 27.3μm to 307 ± 57.2μm and 108 ± 34.2μm to 614 ± 13.1μm, respectively (Fig 3N, 3O, 3Q and 3R). Similarly, SKOV-3_adh_ spheroids grew in diameter after repeated passaging from 139.5 ± 10.6μm to 208.6 ± 87.8μm (Fig 3U and 3X). In contrast, no viable cells or spheroids were detected in SKOV-3_imm_ cells after 3 passages (Fig 3W).

MOSE-L_TIC*v*_ cells, representing fast-developing disease, exhibited a more rapid spheroid forming capacity in response to FSS as indicated by a larger spheroid size. Spheroids grew in diameter after 288 h from 417 ± 97μm to 463 ± 81.4μm and 443 ± 176μm to at least 700μm for MOSE-L_TIC*v*-imm_ and MOSE-L_TIC*v*-adh_ respectively (Fig 3Z, 3AA, 3CC and 3DD). MOSE-L_TIC*v*-adh_ and SKOV-3_adh_ spheroids were able to re-attach to the culture dishes with a moderate (SKOV-3) or large (MOSE-L_TIC*v*_) adherent monolayer outgrowth (Fig 3U, 3X, 3AA and 3DD).

MOSE-L_TIC*v*_ cells formed the largest spheroids observed with an aggressive, multi-layer cell outgrowth already apparent after 96 h (Fig 3Z and 3AA), but even more so after 3 passages (Fig 3CC and 3DD) when spheroids were too large to capture and measure accurately. This outgrowth capacity was lost after the third round of continued exposure to FSS in MOSE-L_TIC*v*-imm_ and SKOV-3_imm_ cells but was maintained in MOSE-L_TIC*v*-adh_ and SKOV-3_adh_ cells. While the increased size of the spheroids was associated with a loss of viable cells in MOSE-L and MOSE-L_TIC*v*_ spheroids after three passages when compared to 96 h time point (see Fig 2), the number of viable cells was maintained in the SKOV-3_adh_ cells. This decrease in viability in the MOSE-L and MOSE-L_TIC*v*_ cells may be due to the loss of adherent cells growing in monolayers over time. In addition, the smaller size of SKOV-3 cells is consistent with previous reports that SKOV-3 cells typically form smaller spheroids than MOSE cells [19, 34].

### 3.3 Cellular architecture is altered by fluid shear stress

Recent evidence suggests that mechanotransduction of extracellular signals into intracellular signals is affected by the organization of the cytoskeleton. In particular, the actin cytoskeleton and its regulators respond to mechanical stimuli and affect cell motility and cellular signaling [35–37]. Thus, we investigated how FSS affects the cytoskeletal architecture of cells at various stages of disease.

**Fig 4:**
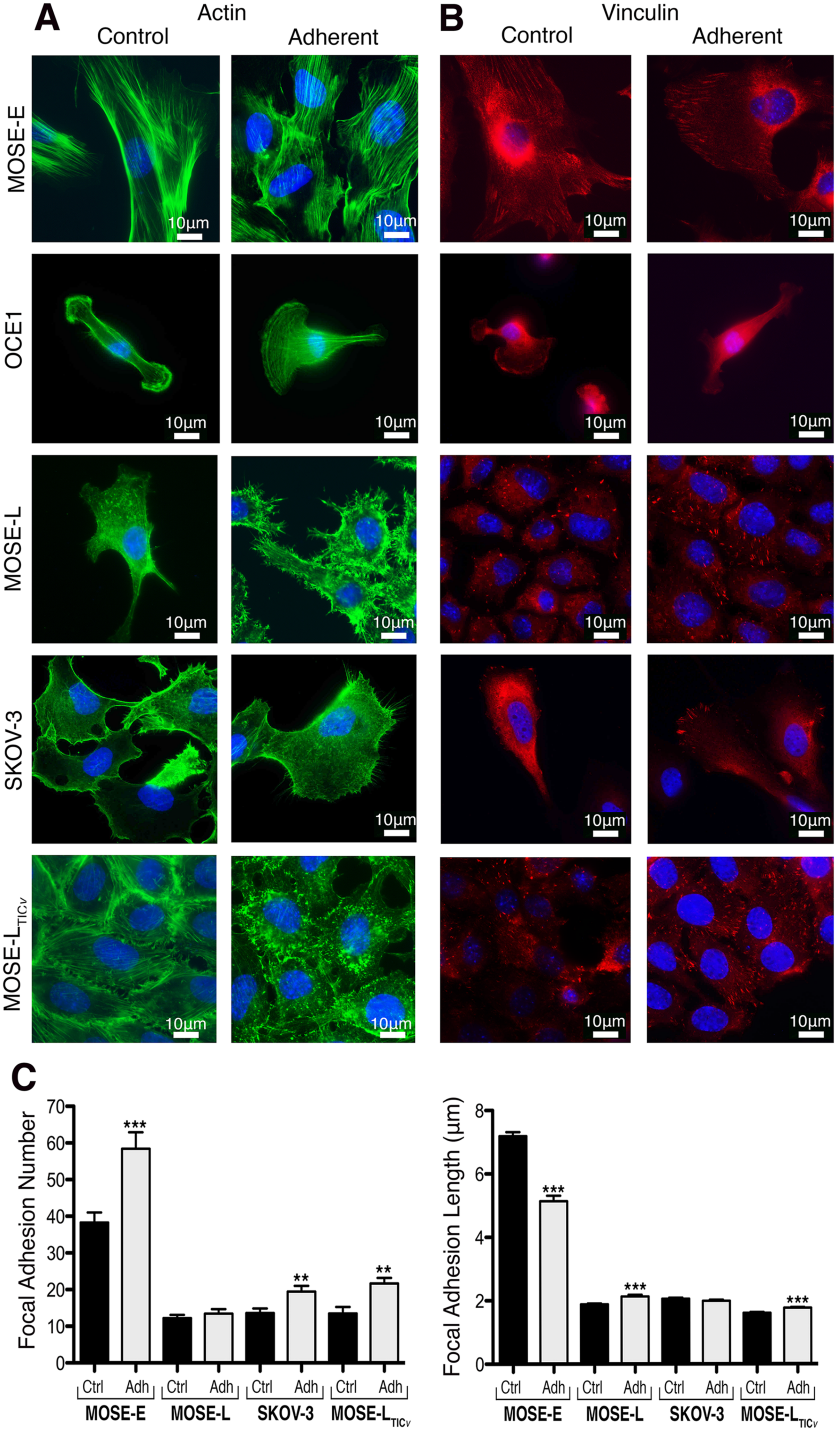
Actin cytoskeleton and focal adhesion organization. Actin cytoskeleton organization and focal adhesion number and length change significantly in response to FSS exposure. (**A**) Changes in actin (green) organization in adherent MOSE-E, COE1, MOSE-L, SKOV-3, and MOSE-L_TIC*v*_ cells after three, consecutive 96 h exposures to FSS. (**B**) FSS effects on vinculin-positive focal adhesions. Nuclei are shown in blue. (**C**) Quantitation of focal adhesion number and size in controls and after FSS exposure. Asterisks denote statistical significance (t-test, * p< 0.05 and *** p< 0.001).

As reported previously [19], MOSE-E cells contain prominent, well defined actin bundles (Fig 4A); in response to the exposure to FSS for 96 h, the actin fibers were found aligned parallel to each other (Fig 4A), comparable to physiological responses of endothelial cells to FSS [38, 39]. OCE1 cells did not adhere sufficiently to the coverslips to allow for a distinct actin or vinculin organization. The actin cytoskeleton in MOSE-L, SKOV-3 and MOSE-L_TIC*v*_ is characterized by thin and short actin fibers and extensive actin-containing protrusions in MOSE-L_TIC*v*_ cells (Fig 4A). Actin protrusions from the cell surface were drastically increased in all tumorigenic cells after exposure to FSS (Fig 4). Later time points were not examined due to the lack of viable monolayers.

Integrin-containing complexes as well as other surface proteins mediate shear stress responses of the cells via modulation of actin bundles and the recruitment of downstream proteins such as vinculin [40]. Thus, in order to begin investigating the FSS-induced changes in cell adhesion, we determined the number and size of vinculin-containing focal adhesions. As shown in Fig 4B and 4C, the benign MOSE-E cells exhibit long focal adhesions that significantly increase in number but decrease in size after FSS exposure. The tumorigenic cells lines (MOSE-L, SKOV-3, and MOSE-L_TIC*v*_) contain fewer focal adhesions, confirming our previous report [24]. There was a small but significant increase in the number of focal adhesions in MOSE-L and MOSE-L_TIC*v*_ cells, and also a significant increase in focal adhesion length in SKOV-3 and MOSE-L_TIC*v*_ cells (Fig 4B and 4C). This suggests that cells respond to FSS with an increase in focal adhesion assembly; however, focal adhesion length and numbers varied in different cells of the same cell line and even cells with very few visible focal adhesions were still attached. Therefore, it seems unlikely that changes in focal adhesions are the sole reason that the adherent cells withstand the forces of the FSS.

### 3.4 Fluid shear stress induces aberrant nuclear morphology and chromosomal instability

**Fig 5:**
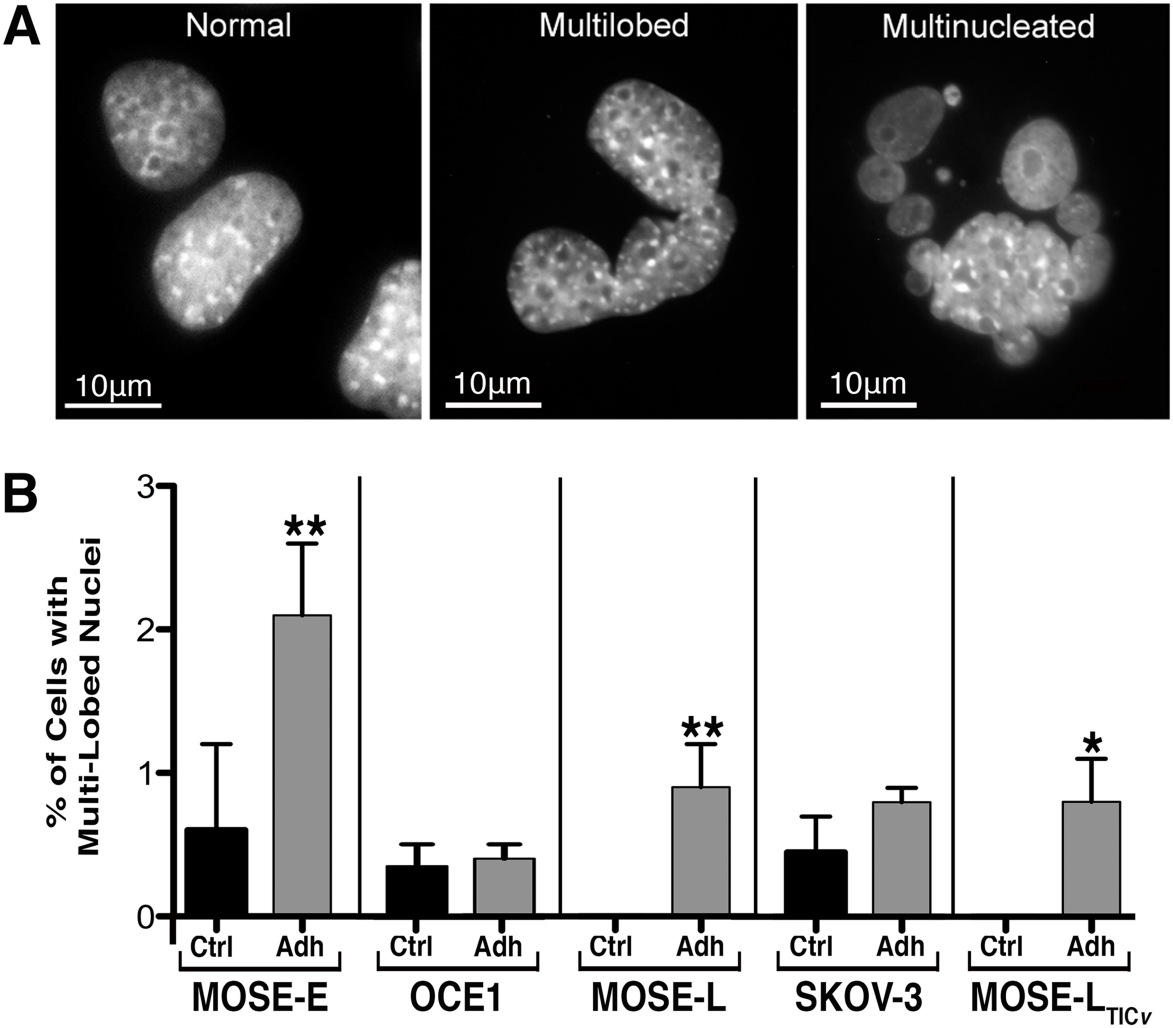
Lobed nuclei images and quantification. FSS induces the emergence of cells with multi-lobed nuclei. (**A**) Representative images of normal nuclei (left), a multi-lobed nucleus (middle), and a multi-nucleated cell (right) observed after three consecutive 96 h (288 h total) exposures to FSS. (**B**) Percentage of cells that exhibit multi-lobed or multi-nucleated nuclei (mean ± SEM). Asterisks denote statistical significance (Fisher’s Exact, * p< 0.05, ** p< 0.005) for comparison to the corresponding control.

The comparably high viability of adherent MOSE-E cells (Fig 2A) and OCE1 cells (Fig 2B) suggested a relative resistance of the benign cells to FSS. However, we observed abnormalities in the nuclear morphology of the cells even prior to FSS exposure. Indeed, low frequencies of multi-lobed nuclei and multi-nucleated cells (collectively referred to as multi-lobed hereafter; Fig 5A) were observed in the benign MOSE-E and OCE1 control cells (0.6% ± 0.3% and 0.35% ± 0.05% respectively; Fig 5B), possibly reflecting the propensity of these cells to acquire abnormal, and eventually tumorigenic, phenotypes during *in vitro* culture over time. In the tumorigenic cell lines, only the SKOV-3 control cells exhibited a smaller percentage of multi-lobed cells (0.45% ± 0.25%). FSS exposure increased the number of multi-lobed cells in all cell lines (Fig 5B, not statistically significant in the SKOV-3 cells) except the benign OCE1 cells.

**Fig 6:**
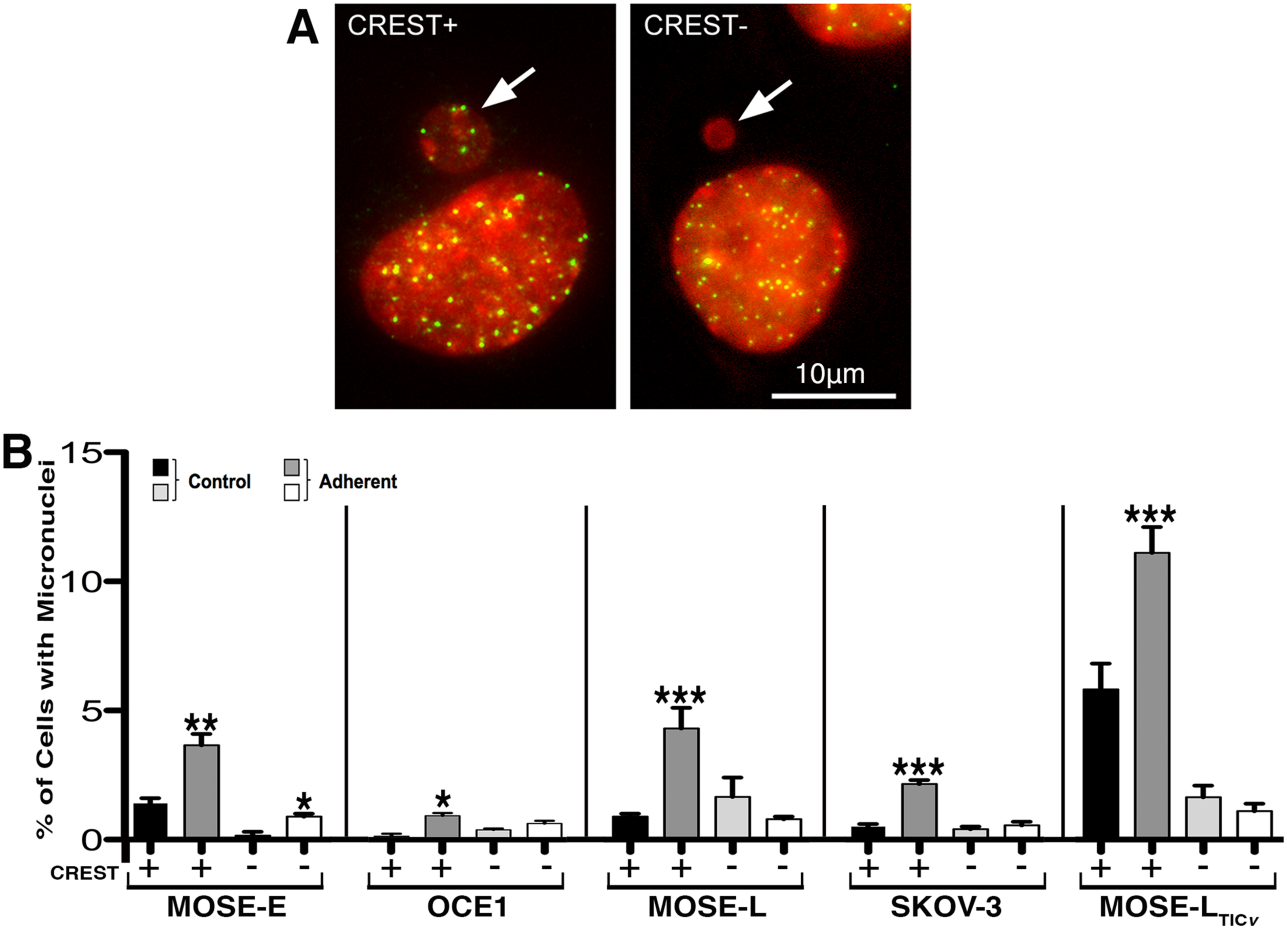
Micronuclei images and quantification. FSS exposure results in an increase in CREST-positive micronuclei. Micronuclei were observed in all cell populations and quantified on multiple slides using CREST (green) and DAPI (red) staining for control and adherent cell populations (after three, consecutive, 96 h exposures to FSS). (**A**) Representative images of cells with either a CREST-positive (CREST+, containing whole chromosomes, left) or a CREST-negative (CREST-, containing chromosome fragments, right) micronucleus (white arrows). (**B**) Average percentages of micronuclei ± SEM. Importantly, all stages of the disease exposed to FSS displayed a significant increase in CREST-positive (whole chromosome-containing) micronuclei. Asterisks denote statistical significance (Fisher’s Exact, * p< 0.05, ** p< 0.005, *** p< 0.001) for comparison of adherent cells exposed to FSS to corresponding controls.

We performed a micronucleus assay in combination with CREST staining [41], which allowed for the discrimination of micronuclei arising from whole chromosome mis-segregation (CREST-positive; Fig 6A, left panel) from micronuclei arising from DNA fragments (CREST-negative; Fig 6A, right panel). Low levels of CREST-positive micronuclei were observed in cell lines not treated with FSS but a significantly (p< 0.05) higher percentage of CREST-positive micronuclei was found in the control MOSE-L_TIC*v*_ cells (5.8% ± 1%) compared to the MOSE-E (1.4% ± 0.2%), OCE1 (0.1% ± 0.1%), MOSE-L (0.6% ± 1%), and SKOV-3 cells (0.5% ± 0.1%). After exposure to prolonged FSS, a significant increase in CREST-positive micronuclei was observed in all cell lines. This increase in CREST-positive micronuclei was more pronounced in the murine cell lines with up to 5% of the population in MOSE-E (p< 0.005) and MOSE-L (p< 0.001) and 11% in MOSE-L_TIC*v*_ (p< 0.001) than in the human cell lines where the increase only rose to 1% of the population in OCE1 (p< 0.04) and over 2% in SKOV-3 (p< 0.001) cells. Low levels of CREST-negative micronuclei were observed in control MOSE-E (0.15% ± 0.15%), OCE1 (0.35% ± 0.05%), and SKOV-3 (0.4% ± 0.1%) cells as compared to MOSE-L (1.65% ± 0.75%) and MOSE-L_TIC*v*_ (1.65% ± 0.45%) cells. Only MOSE-E cells exposed to FSS also included a significant increase in CREST-negative micronuclei to the levels comparable to those observed in the murine tumorigenic lines (Fig 6B).

**Fig 7:**
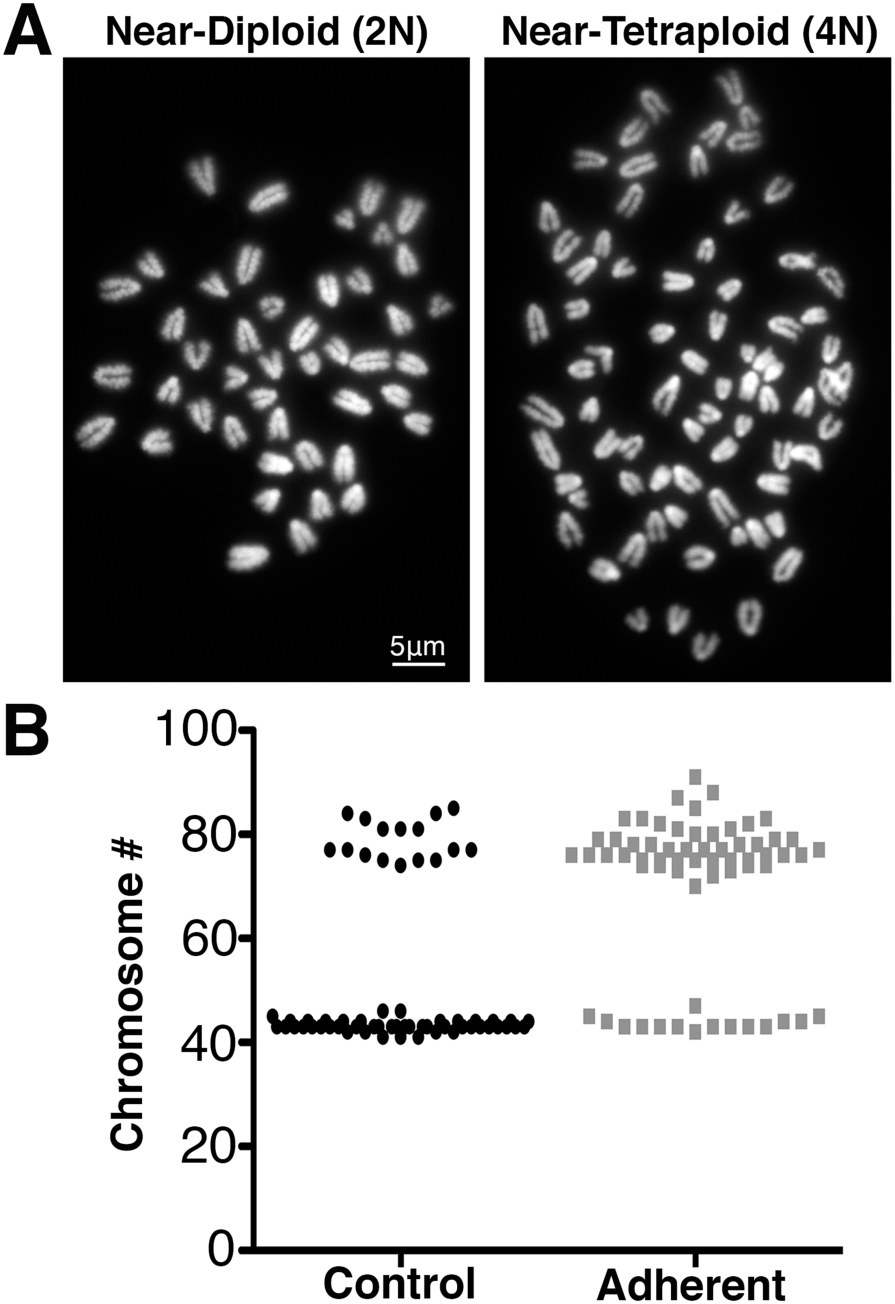
Metaphase images and quantification. FSS dramatically increases the fraction of MOSE-E cells with near-tetraploid chromosome numbers. (**A**) Examples of metaphase spreads with near-diploid (left) and near-tetraploid (right) range. (**B**) Chromosome counts in individual metaphase spreads from the control (black) and FSS-exposed adherent (gray) MOSE-E cells. Upon exposure to FSS, the fraction of near tetraploid cells increased to 76% ± 2.4% compared to 31% ± 5.0% in the control population (*χ*^2^, p = 0.013).

The presence of multi-lobed nuclei and CREST-positive micronuclei suggested that FSS may cause errors in chromosome segregation during cell division, which would be apparent as changes in chromosome numbers in the cell population. This change would be especially important in benign cells, in which changes in chromosome number could contribute to the establishment of a transformed phenotype [42]. To assess the emergence of chromosome number variation, we counted chromosomes in metaphase spreads (Fig 7) prepared from control MOSE-E cells and FSS-exposed MOSE-E cells.

Under both control and FSS conditions, we found two subpopulations with modal chromosome numbers in the near-diploid (≈ 40) and the near-tetraploid (≈ 80) range (Fig 7A). The presence of a small tetraploid subpopulation in MOSE-E cells is consistent with previous reports [19, 43]. In response to FSS exposure, the near-tetraploid subpopulation became significantly (p = 0.013) larger and came to represent a much larger fraction of the population than what is observed in the control (76% ± 2.4% versus 31% ± 5.0%). Moreover, the MOSE-E_adh_ near-tetraploid subpopulation displayed larger chromosome number heterogeneity compared to the MOSE-E near-tetraploid subpopulation (Fig 7B). Indeed, the near-tetraploid MOSE-E control cells had chromosome numbers ranging between 74 and 85, whereas for the near-tetraploid MOSE-E_adh_ cells, the chromosome number ranged between 70 and 91. These data suggest that FSS may promote the generation of tetraploid cells by inducing defective cell division. Alternatively, the increase in the near-tetraploid sub-population may be due to a biased survival of tetraploid cells as compared to the diploid cells under FSS, leading to an increase of the near-tetraploid fraction of the population.

## 4 Discussion and conclusions

All organs of the body are exposed to different mechanical stresses that are critical for the organization, differentiation and function of cells such as endothelial, osteoblast and renal tubular cells [35–37], while the same mechanical stresses are detrimental for epithelial cells [44, 45]. Here, using the estimated magnitude of ascites motion in the peritoneal cavity, we investigated the impact of FSS on murine and human ovarian cells ranging from a non-transformed cell type to a transformed, highly-aggressive cell type, representing the increasing aggressiveness of progressive ovarian cancer. All cells were subjected to repetitive, 96 h periods of FSS of magnitudes less than $1\frac{dyne}{cm^{2}}$ with two exposure types that mimicked disseminated cells (imm groups) and attached cells of the ovarian lining (adh groups). This experimental design allowed us to investigate cells over prolonged exposure and potentially enhanced the percentage of the population with altered properties for analyses. This re-plating may also represent physiologic events. Indeed, it is highly possible that FSS in the peritoneal cavity causes exfoliation of surface cells, which could then re-adhere at a different location. The lab rotator system chosen for our analyses produces a swirling flow we consider to be a reasonable approximation of the peritoneal fluid motion generated by diaphragm movements [5]. Although higher levels of FSS may occur in vivo in more advanced stages of the disease our data show that even very low levels of FSS is sufficient to negatively impact cells. Overall, we observed that FSS differentially impacted the viability of mouse and human cells. All cells exhibited a significantly reduced viability when exposed to FSS before adherence but only the slow-developing disease (MOSE-L) also showed a significant loss of viability when exposed to FSS in an adherent state; all other cells showed some resistance to FSS. FSS induced events that are associated with a more aggressive and metastatic phenotype including an increasing capacity for spheroid formation of the tumorigenic cell lines, the disorganization of actin stress fibers and the development of a multitude of actin-containing protrusions, and an increase in vinculin-containing focal adhesions. Importantly, nuclear changes such as an increase in the frequency of multi-lobed nuclei and CREST-positive micronuclei in response to FSS in all cell types and the increased number of benign cells with tetraploid chromosome number demonstrate that even low magnitudes of FSS affect the phenotype of cancer cells and can induce changes in the benign ovarian epithelial cells with potentially severe consequences.

The sensitivity to FSS and the significant reduction of viability over time we observed in both the mouse and human cancer cells used in this study were comparable to the effects observed in established cancer cell lines, although this previous study used FSS of higher magnitudes [46]. Also, the apparent resistance to FSS and subsequent survival of adherent benign MOSE-E cells we observed is comparable to that described in benign NL20, CHL [46], hematopoietic [47], and endothelial [46] cells. In contrast, Barnes et al. found a higher resistance of transformed cells compared to benign cells after exposure to FSS but found no difference among cancer cell lines characterized by different aggressiveness levels [45]. Differences in the experimental design, such as duration and magnitude of exposure (cells were exposed to a similar magnitude of FSS, but only for 12-48h in the study by Lien et al. [46]; cells were exposed to up to $6,400\frac{dyne}{cm^{2}}$ of FSS for short-term durations by Barnes et al. [45]), culture techniques (adherent cells versus non-adherent cells) and the cell type (ovarian epithelial cells in our study versus established cancer cells lines, benign lung cancer cells, and endothelial cell lines, which physiologically require exposure to FSS for organization and function) likely account for the different phenotypes observed in response to FSS. Differences in the response could also be attributable to differences in doubling rate (OCE1 and SKOV-3 have a doubling rate of > 35 h compared to the MOSE cells < 22 h).

Spheroid formation has been associated with increased invasive capacity [32], resistance to drug treatment [33, 48], stem cell phenotypes [48], and increasing resistance to apoptosis and higher metastatic potential of the spheroids [31]. This invasive capacity after aggregation is not specific to ovarian cancer, as the aggregation of breast cancer cells has been shown to increase their metastatic potential by 20-50 fold [31]. Furthermore as observed previously, pre-formed spheroids of SKOV-3 cells increased expression of stemness markers after exposure to FSS [48]. In the present study, all tumorigenic cell lines formed large, viable spheroids upon FSS exposure, capable of re-attaching to the culture dish and growing out in monolayers or as multilayered, more invasive structures as observed in the the MOSE-L_TIC*v*_ cells (Fig 3). These results are comparable to physiological conditions in the peritoneal cavity, where ovarian cancer cells disseminate either as single cells or as spheroids [6] of varying sizes. Spheroid formation is diminished in established cell lines; the formation of only small spheroids in human ovarian cancer cell lines with lower viability have been reported [18]. However, while the FSS-induced SKOV-3 spheroids were smaller, they were viable and grew over time and their adhesion capacity was more comparable to the MOSE-L_TIC*v*_ cells. Both cell lines are derived from ascites, and their tumorigenic potential may be reflected by their capacity to adhere after spheroid formation and grow out in multi-layered structures. These observations indicate that our experimental design is well suited to mimic fluid flow processes that occur under physiological conditions and both murine and human cells respond in similar fashion. Thus, the FSS-induced aggregation of cancer cells and prolonged exposure of these spheroids to FSS may contribute to enhanced metastatic capacity of the cancer cells. The molecular mechanisms underlying these changes warrant further investigation.

In addition to spheroid formation, we observed drastic changes in cytoskeleton arrangement and focal adhesion assembly in response to FSS. Cellular architecture has been identified as a critical factor for the transduction of extracellular signals to intracellular signals [49, 50]. Disruption or alterations in key cytoskeletal components like actin can impact mechanotransduction of signals, and alter the cellular responses. We have previously shown that changes in the cytoskeletal architecture during MOSE progression affects the spatial localization of critical signaling intermediates such as PKC*β*II [24], a kinase with a broad spectrum of targets; changes in its localization could affect which signaling pathways are activated, and, thus, alter the responses of cells. Actin dynamics have been shown to allow for the formation of micro-domains that recruit signaling proteins, and, thereby, direct signaling pathways [51]. This has also been shown for endothelial and smooth muscles cells that respond to physical stress with actin fiber alignment, and the recruitment of specific proteins to the actin fibers [52]. It is possible that the parallel organization of actin fibers observed in MOSE-E cells and OCE1 cells after exposure to FSS could also impact signaling events. While no fiber alignment was observed in the tumorigenic MOSE or SKOV-3 cells, they exhibited extensive actin-containing protrusions which have been associated with increased invasiveness and mechanosensitivity (see recent reviews [53, 54]). These were not observed in the benign cells, suggesting that these actin protrusions could be invadopodia [55] expressed solely in metastatic cells that support invasion by extracellular matrix degradation and remodeling [54] (punctae in MOSE-L_TIC*v*_, Fig 4F) and filopodia that are involved in extracellular matrix sensing and motility [56] more apparent in MOSE-L (Fig 4D). These protrusions may provide the tumorigenic MOSE and SKOV-3 cells enhanced metastatic capacities. Focal adhesions link the extracellular matrix to the actin cytoskeleton, and are critically involved in mechanosensing. Vinculin is recruited to the focal adhesions in response to mechanical stress and has been associated with the strengthening of adhesions [57]. This effect has been shown to be force dependent [40]. However, even the low magnitude of FSS imposed onto the cells in the present study resulted in the assembly of vinculin-containing focal adhesions, likely contributing to the cells adhesion capability. It is unclear if the cells that did not adhere during FSS treatment failed to enhance their focal adhesions, or if other, additional mechanisms are contributing to a stronger adhesion.

The loss of viability of non-adherent benign MOSE-E and OCE1 cells after prolonged exposure to FSS suggests that benign cells or cells with low metastatic potential exfoliated into the peritoneal cavity may not survive in high numbers; however, the cells that do survive may exhibit a more malignant phenotype. All cells that survived exposure to FSS, independent of their disease stage, exhibited an increase in the frequency of multi-lobed nuclei and micronuclei over time, suggesting a selection for these cells during passaging. A particularly high increase in CREST-positive micronuclei in response to FSS was found for all cell types. CREST-positive micronuclei are known to originate from chromosome segregation errors during mitosis [58, 59], indicating that FSS can affect the fidelity of cell division and could trigger events that lead to aneuploidy (incorrect chromosome number), a well-recognized hallmark of cancer [42, 60]. Moreover, DNA enclosed in micronuclei has been shown to accumulate damage due to defects in DNA repair and replication (reviewed in [61]), leading to accumulation of chromosome structural rearrangements, another common feature of cancer cells. Importantly, while murine cells spontaneously immortalize and transform over time (our MOSE model), human ovarian surface epithelial cells have a definite number of cell divisions and subsequently undergo senescence; transfection with telomerase for immortalization (OCE1 cells), or human papillomavirus type 16 is required to allow for transformation [62], indicating a more stable genome in human cells than in mice. While the increase of nuclear aberrations in the human cells was less than observed in murine cells, FSS also significantly increased micronuclei containing whole chromosomes, indicating that even in a more stable genome is not resistant to the detrimental effects of FSS.

Analysis of chromosome number in individual cells revealed yet another genomic change, tetraploidization (Fig 7), likely arising from defective cell division in MOSE-E cells exposed to FSS. Importantly, tetraploidy is found in a number of premalignant lesions [63, 64], has also been shown to promote tumorigenesis [65], and arises spontaneously during tumorigenic progression of MOSE cells *in vitro* [43, 66]. Thus, our results show that, by increasing the rate of tetraploidization in benign ovarian cells, FSS may promote or contribute to the establishment of a transformed phenotype, linking biophysical forces to carcinogenesis.

Overall, our results indicate that even a low level of continual FSS significantly and differentially affects adherent epithelial ovarian cancer cells of various stages of progression. In particular, benign cells that survive under FSS display phenotypic and genotypic changes that can be associated with malignant with a premalignant conditions. These observations fit well into a cancer evolution model proposing that various stresses (internal such as mutations and oncogenes, external such as physical stresses, and experimental manipulations such as knock-outs or transfections) result in genome instability and lead to population diversity that drives tumorigenicity. This is a genome- but not gene mutations-driven process and, thus, independent of specific mutations [1]. The observed genomic changes in FSS-treated cells therefore could be a contributing factor not only to the progression of ovarian cancer but also to the transformation of benign cells; the increased adhesion of these cells may not be an advantage since it will ensure the presence of cell with a more transformed phenotype and may link peritoneal inflammation with ascites to cancer risk.

## Supporting information

S1 Dataset Raw data. All raw data from this work can be found here.
(XLSX)
